# Author Correction: Selective Priming of Tumor Blood Vessels by Radiation Therapy Enhances Nanodrug Delivery

**DOI:** 10.1038/s41598-020-72253-7

**Published:** 2020-09-15

**Authors:** Sijumon Kunjachan, Shady Kotb, Robert Pola, Michal Pechar, Rajiv Kumar, Bijay Singh, Felix Gremse, Reza Taleeli, Florian Trichard, Vincent Motto-Ros, Lucie Sancey, Alexandre Detappe, Sayeda Yasmin-Karim, Andrea Protti, Ilanchezhian Shanmugam, Thomas Ireland, Tomas Etrych, Srinivas Sridhar, Olivier Tillement, Mike Makrigiorgos, Ross I. Berbeco

**Affiliations:** 1 Department of Radiation Oncology, Brigham and Women’s Hospital, Dana-Farber Cancer Institute and Harvard Medical School, Boston, MA United States; 2 Institut Lumière Matière, UMR 5306, Université Claude Bernard Lyon 1, CNRS, Villeurbanne, France; 3 Nanomedicine Science and Technology Center and Department of Physics, Northeastern University, Boston, MA United States; 4 Institute of Macromolecular Chemistry, Academy of Sciences of the Czech Republic, Heyrovsky Square 2, 16206 Prague 6, Czech Republic; 5 Experimental Molecular Imaging, University Hospital and Helmholtz Institute for Biomedical Engineering, RWTH Aachen University, Aachen, Germany; 6 Division of Medical Physics & Engineering, University of Texas Southwestern Medical Center, Texas, United States; 7 Institute for Advanced Biosciences, UGA/INSERM U1209/CNRS UMR 5309 Joint Research Center, Grenoble, France; 8 Lurie Family Imaging Center, Department of Radiology, Dana-Farber Cancer Institute and Harvard Medical School, Boston, MA United States; 9 LA-ICP-MS and ICP-ES Laboratories, Department of Earth and Environmental Sciences, Boston University, Boston, MA United States

Correction to: *Scientific Reports* 10.1038/s41598-019-50538-w, published online 01 November 2019

This Article contains errors. For Figure 2E, a cell from the 4 Gy without t-NPs condition was inadvertently duplicated for the 2 Gy, with t-NPs, panel. In addition, the image for 2 Gy w/o t-NP in the original article is incorrect. A corrected version of Figure 2E appears below as Figure 1, with raw images rather than the enlarged composites that had been used in the original Article.

**Figure 1:**
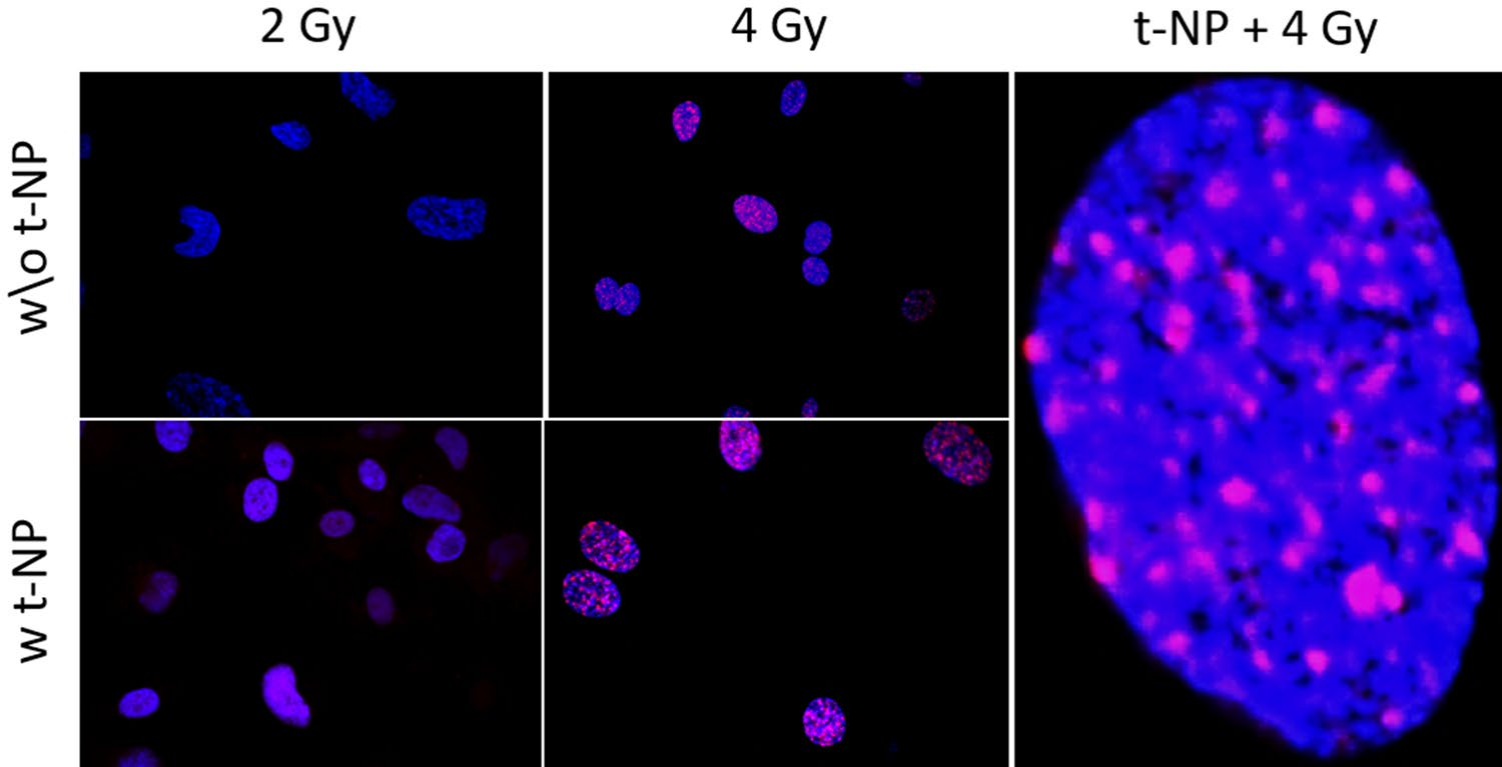
A corrected version of Figure 2E in the Article.

An error in the assembly of Supplementary Figure S3 resulted in duplication of several images. We have repeated this experiment, and the new data are presented below as Figure 2.

**Figure 2:**
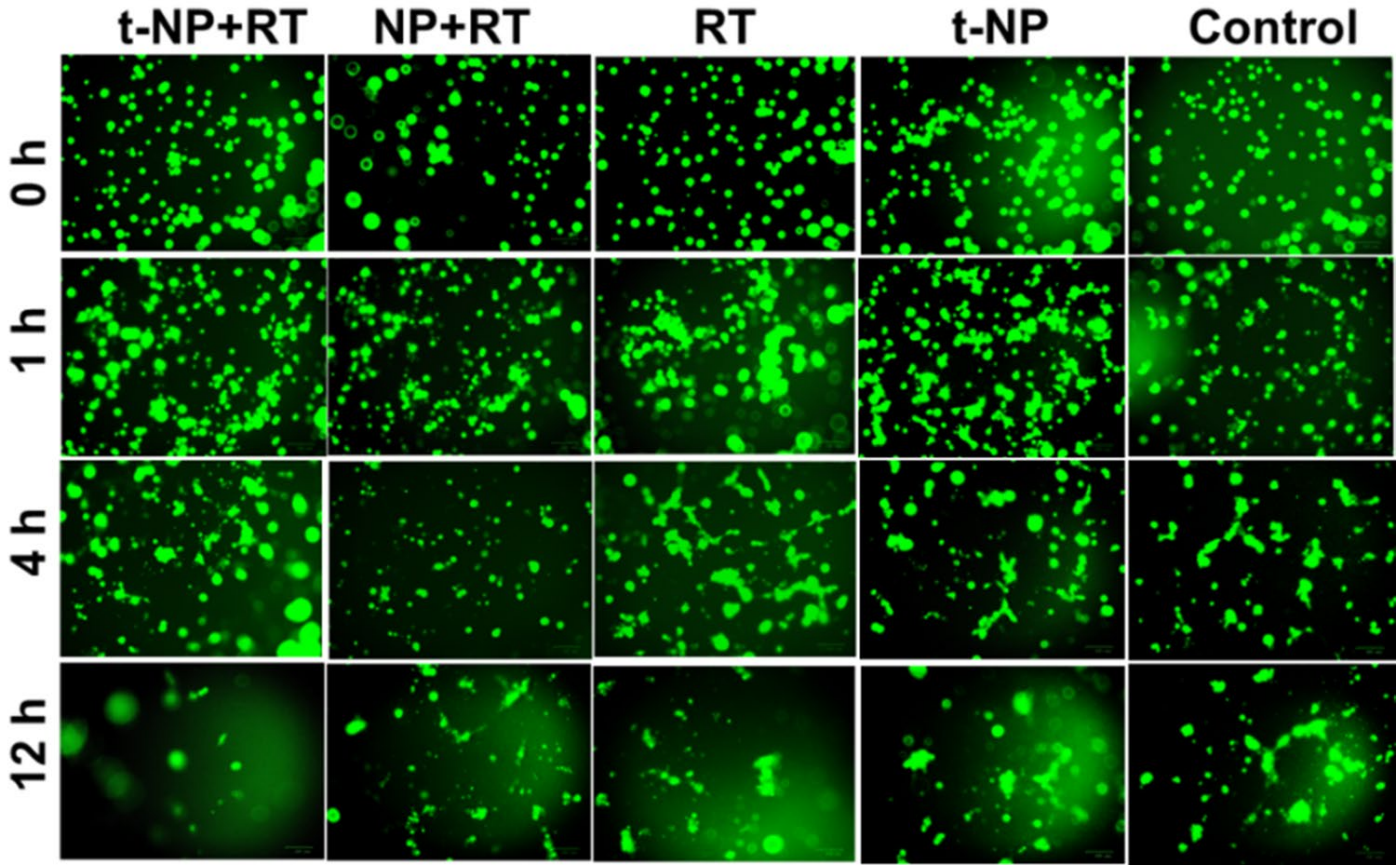
A corrected version of Supplementary Figure S3 in the Article.

For Figure S8, the control images for the heart and lung were misidentified as belonging to the treatment group. In addition, in reassessing this data, we could not validate the image for the control condition of the bladder. The corrected Figure S8 includes the correct images for each condition, and is presented here as Figure 3.

**Figure 3:**
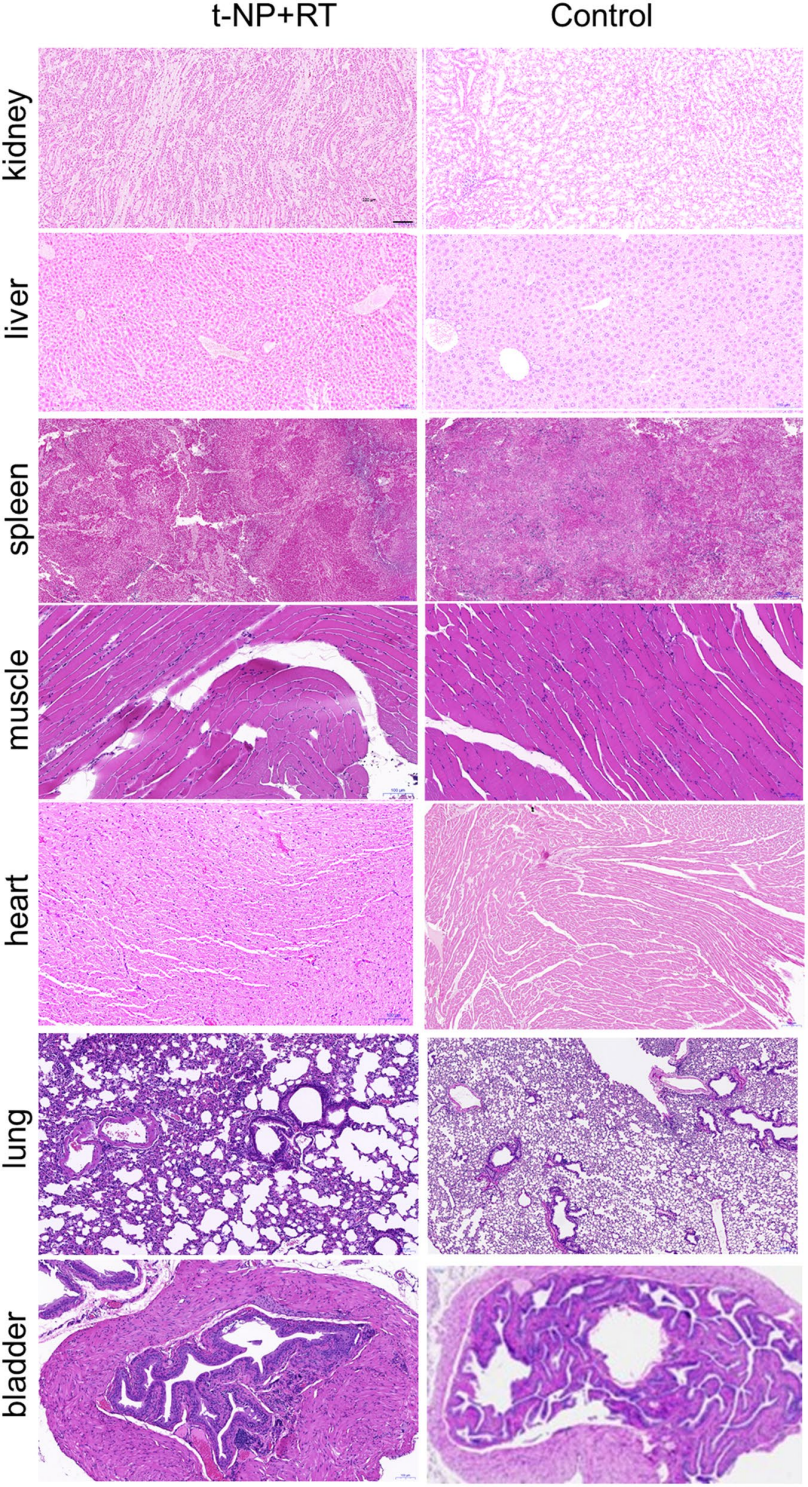
A corrected version of Supplementary Figure S8 in the Article.

These corrections do not change the conclusions of the paper.

